# On-Court Activity and Game-Related Statistics during Scoring Streaks in Basketball: Applied Use of Accelerometers

**DOI:** 10.3390/s22114059

**Published:** 2022-05-27

**Authors:** Jodie A. Palmer, Rodrigo Bini, Daniel Wundersitz, Michael Kingsley

**Affiliations:** 1 Holsworth Research Initiative, La Trobe Rural Health School, La Trobe University, Bendigo, VIC 3552, Australia; jodie.palmer@latrobe.edu.au (J.A.P.); r.bini@latrobe.edu.au (R.B.); d.wundersitz@latrobe.edu.au (D.W.); 2 Department of Exercise Sciences, Faculty of Science, University of Auckland, Auckland 1023, New Zealand

**Keywords:** coaching, game analysis, performance, tactics, accelerometry

## Abstract

The aim of this observational study was to determine if on-court activity and match statistics differed between periods of scoring streaks and regular play in basketball. Thirty-seven basketballers including professional women, semi-professional women and semi-professional men wore accelerometers during competitive matches throughout a season. Accelerometry-derived live-time individual on-court exercise intensity and team game-related statistics were compared between scoring streaks (periods of play where the teams participating in the study scored at least three times in a row), streaks against (periods of play where the opposition teams scored at least three times in a row) and regular play. Few differences existed in the average exercise intensity between streak types. During streaks against, there was a 5–15% lower proportion of 2-point attempts, 0.8–1.3 fewer defensive rebounds per minute and 0.3–1.6 fewer shot attempts per minute compared to regular play and scoring streaks, and there were 0.3 fewer offensive rebounds per minute compared to regular play. During scoring streaks, there were 0.5 more defensive rebounds per minute, 1.3 more shot attempts per minute, a 43% greater shooting percentage and a 10% lower proportion of 3-point attempts compared to regular play. To reduce the chances of streaks against, teams should focus on facilitating 2-point shot attempts and consider implementing a 3:1 ratio of 2-point to 3-point attempts to maximize scoring success, and they should focus on winning rebounds to facilitate more shot attempts.

## 1. Introduction

The match analysis of physical demands and game-related statistics has been shown to explain differences in match outcome and identify key performance indicators for success [1,2,3,4,5,6,7]. Knowledge of these key performance indicators can guide training prescription and strategy to improve the overall success of the team. For example, analysis of team game-related statistics in elite men’s and women’s basketball shows that field goal percentage, offensive and defensive rebounds, assists, steals, blocks and fouls against are greater, while turnovers and fouls committed are lower, in wins compared to losses [1,2,3,4]. On-court match demands can be influenced by various situational and contextual factors, such as playing position [8], playing period [8] and match outcome [6,7,9]. When exploring physical match demands between wins and losses, findings differ between studies. For example, in semi-professional men’s basketball, Fox et al. (2019) reported that starters performed more jumps, more absolute and relative high-intensity accelerations and decelerations and more relative changes of direction during losses, whereas perceived exertion was higher during wins. However, no differences were identified in PlayerLoad™, accelerations, decelerations, changes of direction, jumps or heart rate when comparing winning and losing quarters in competitive matches [6]. In professional men’s basketball, when assessing 10 min match simulations rather than whole competitive matches, players covered greater high-speed running distances in wins than in losses [9]. A limitation of previous studies investigating time-motion analysis variables is that match activities have been assessed as total time, including all stoppages and substitution breaks. Because match activity is strongly influenced by how much time a player spends on the court [10], and any activity while substituted or when the ball is not in play does not directly contribute to the match outcome, it is logical to consider only on-court physical activity during live time when assessing the associations between physical demands and match performance.

While it is conceivable that the inconsistencies in the findings for on-court activity between wins and losses are due to contextual factors (i.e., comparing whole competitive matches with 10 min match simulations and match quarters), it is also possible that assessing performance outcomes for the match or quarter as a whole does not enable the detection of differences between optimal and sub-optimal performance. Changes in scoring momentum occur frequently in sport, exhibiting an abnormal enhanced scoring progression by one team, typically characterized by a noticeable shift in performance [11,12]. The presence of such periods of scoring momentum indicates that performance is often transient within a match or a quarter. Investigating the differences between periods of optimal and sub-optimal performance within a match might, therefore, uncover trends not detected when assessing the overall performance of a match or quarter.

A challenge of investigating factors associated with scoring momentum is the abstract nature of the concept of momentum in sport [12]. While the concept of scoring momentum in sport is familiar to coaches, players and spectators [13], no consensus currently exists around which parameters define a period of scoring momentum. In basketball, as possession changes after a scoring event, the probability of the opposition team scoring next is almost double the probability of the same team scoring again [14]. Therefore, successive scoring events for the same team can be considered abnormal, and scoring streaks can therefore be considered as periods of positive scoring momentum for the streaking team and as negative scoring momentum for the opposition. Knowledge of the factors associated with scoring streaks might assist coaches to make strategic decisions to maximize scoring streaks and minimize streaks against their team.

Previous researchers investigating scoring momentum in sport have focused on potential psychological influences (e.g., [11,15,16]). Positive and negative scoring momentum in sport has been associated with changes in emotion, behavior and cognition [16]. Such factors include motivation, perceptions of control, energy and team synchronization [15]. It has been suggested that scoring streaks or periods of positive scoring momentum can be generated by a precipitating event, such as a dramatic play or an important player leaving the match for a negative reason [11]. It has also been suggested that time-outs can be effective in stopping an opposition team’s scoring momentum [17]. While this information can be useful for coaches to detect potential catalysts for scoring streaks and provide a tool for limiting the opposition’s scoring momentum, knowledge of the physical and tactical factors that contribute to scoring streaks occurring could enable coaches to maximize scoring streaks and minimize streaks against their team without having to use a time-out. For example, if scoring streaks are associated with a specific match event (e.g., rebounds), rather than calling a time-out to stop the opposition team’s streak, a coach could instruct the players to focus on rebounding. Additionally, while individual-based scoring streaks (‘hot hand’; [18,19,20,21]) have been investigated extensively, to the best of the authors’ knowledge, no research has been conducted on game-related statistics or physical activity intensity factors associated with team-based scoring streaks in basketball. Therefore, the aim of this study was to determine if differences in individual on-court activity intensity and team game-related statistics exist when teams are experiencing scoring streaks in comparison to regular play in basketball.

## 2. Materials and Methods

### 2.1. Study Design and Setting

**Figure 1:**
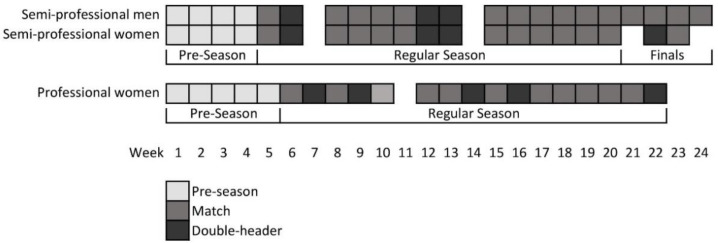
Schematic of the season timelines.

This study was observational. On-court activity intensity and game-related statistics data were collected throughout a single Australian semi-professional men’s, semi-professional women’s and professional women’s basketball season. The players were recruited before the beginning of the pre-season period (February 2019 for the semi-professional teams and September 2019 for the professional team), and the data were collected from the start to the end of the season (August 2019 for the semi-professional teams and February 2020 for the professional team). The season timelines are outlined in Figure 1.

### 2.2. Participants

Using G*Power (v3.1.9.7; Universität Düsseldorf, Düsseldorf, Germany; [22]), an a-priori sample size calculation was completed based on the primary aim of determining if differences in individual on-court activity intensity and team game-related statistics exist between periods when streaks were occurring (scoring streaks and streaks against) in comparison to regular play. A predicted effect size of 0.68 was selected from previous research that showed that high-speed running meters per minute was greater in wins compared to losses in professional basketball [9]. The differences between two dependent means determined that, with a Bonferroni adjusted alpha of 0.017, a power of 0.8 and an effect size of 0.68, a sample size of at least 26 was required.

All players contracted to a local semi-professional or professional basketball team were recruited to participate in the study (*n* = 37). Temporary replacement players were excluded because they did not train with the semi-professional or professional teams, and their participation in matches was transient. The players were recruited from one professional women’s team, one semi-professional women’s team and one semi-professional men’s team (Table 1) from the same basketball organization, where each team was coached by different coaching staff. A professional men’s team was not available. The professional team competed in the Australian 2019/20 WNBL season (the highest level of Australian women’s competition), and the semi-professional teams competed in the Australian 2019 NBL1 season (the second-highest level of Australian men’s and women’s competition). The semi-professional teams trained 1–2 times per week and played 1–2 matches per week on weekends. The professional team trained 2–3 times per week and played 1–2 matches per week on weekends. The players provided written informed consent prior to participating. Ethical approval was granted by the La Trobe University Human Research Ethics Committee (HEC15-088) in accordance with the Declaration of Helsinki.

### 2.3. Procedures

During the pre-season, the players who were injury-free (11 professional women, 7 semi-professional women, 12 semi-professional men) performed a modified Yo-Yo Intermittent Recovery 1 (IR1) test while instrumented with an accelerometer to estimate the average net force output (AvF_NET_) at various walking and running speeds. Seven additional stages of speeds ranging from 3 km∙h^−1^ to 9 km∙h^−1^ were added immediately prior to the standard Yo-Yo IR1 test to encompass slower movement speeds. Stature and mass were also measured during this testing session.

During matches throughout an entire competitive season, the players’ movement activities were monitored with a 100 Hz triaxial accelerometer (GT9X Link; Actigraph, FL, USA) worn in a pouch on a tightly-fitted bib and positioned between the athlete’s scapulae [23]. Accelerometers have shown acceptable intra- and inter-device reliability in laboratory and team sport settings [24,25,26,27] and are preferable for quantifying basketball activity compared to two-dimensional methods such as video analysis [28]. The accelerometer data were downloaded using the manufacturer’s software (Actilife v6.13.4; Actigraph, FL, USA). On-court match activity (average intensity and the proportion of match time spent performing inactive, light, moderate-vigorous, maximal and supramaximal activity) and game-related statistics (fouls, shot attempts, rebounds, shooting percentage and the proportion of scoring shots involving an assist) were compared between periods of scoring streaks, streaks against and regular play.

### 2.4. Data Analyses

The average activity intensity and the time spent in individualized intensity bands were determined using the accelerometry-derived methods described previously [8,10,29]. Briefly, a player’s average resultant acceleration per second was multiplied by their body mass to calculate AvF_NET_, representing their activity intensity. Using established relationships between estimated oxygen consumption (${\dot{V}O}_{2}$) and walking and running speeds [30], individualized relationships between AvF_NET_ and %${\;\dot{V}O}_{2}$ reserve were calculated using each player’s average AvF_NET_ output during each speed of the Yo-Yo IR1 test. These relationships were then used to categorize activity into intensity bands. The intensity bands were defined as inactive (≤10% ${\dot{V}O}_{2}$ reserve), light (>10–40% ${\dot{V}O}_{2}$ reserve), moderate-vigorous (>40–90% ${\dot{V}O}_{2}$ reserve), maximal (>90–100% ${\dot{V}O}_{2}$ reserve) and supramaximal (>100% ${\dot{V}O}_{2}$ reserve), as described previously [10].

As basketball match demands are strongly influenced by minutes played, only the time during which a player was on-court in live play was included in the analyses. The time points representing when each player was on-court and in live play were determined using an accelerometry-derived automated moving average sample window method, which was previously shown to be valid against manually determined time points [28]. Briefly, this method used a 9 s sample window to calculate the moving average activity intensity for each player. Each moving average value was determined to be either above or below an individualized intensity threshold of 31% ${\dot{V}O}_{2}$ reserve. Periods of play where the average intensity across the 9 s sample window was above the threshold were considered as on-court live play for that player. On-court live play activity was then separated from total match activity. The on-court live play activity for each player for each match was then allocated to either scoring streaks, streaks against or regular play. The season-average activity during scoring streaks, streaks against and regular play for each player was used for the analyses.

Play-by-play match reports were obtained from league websites [31,32]. Match events and associated time stamps were extracted from these reports, and instances of scoring streaks were determined using custom MATLAB code (R2018b; MathWorks, Natick, MA, USA). As a definition for scoring momentum or scoring streaks in basketball did not exist prior to this study, a scoring streak was defined as three or more consecutive scoring events for a team at a rate of at least one scoring event per 36 s. The minimum requirement of at least three consecutive scoring events was selected because streaks are generally considered as three successive events [33]. A minimum average scoring rate was included to ensure the streaks characterized in this study were reflective of abnormal play. For the matches included in this study, the median scoring rate for either team to score was one scoring event every 24 s. To eliminate periods of play where three consecutive scoring events occurred due to one team missing their shots without any noticeable shift in performance, the minimum average scoring rate for a streak to be included was one scoring event every 1.5 times the median scoring rate, or every 36 s. This rate of 1.5 times the median scoring rate was selected rather than the median scoring rate due to the median scoring rate being reflective of either team scoring and not encompassing the time required for possession to be regained by the scoring team. Scoring streaks were considered to have ended once the other team scored or at the end of the quarter. As time-outs are commonly used by coaches to stop an opposition team’s momentum [17], the frequency of time-outs called by the opposition during scoring streaks was compared to the frequency of time-outs called during regular play to assess the face validity of the scoring streak definition. Team game-related statistics during scoring streaks, streaks against and regular play were manually extracted from the play-by-play reports for subsequent analyses.

### 2.5. Statistical Analyses

The statistical analyses were conducted using IBM SPSS Statistics for Windows (Version 26.0; IBM Corp., Armonk, NY, USA), with significance set at *p* ≤ 0.05. Shapiro–Wilk tests indicated that several variables violated the assumption of normality. Therefore, the data were log-transformed prior to parametric analysis, and the descriptive data were presented as median (lower quartile–upper quartile). Mixed-model ANOVAs were conducted with streak type (three levels: scoring streaks/streaks against/regular play) as the within-subjects factor and team (three levels: professional women, semi-professional women, semi-professional men) as the between-subjects factor. When the assumption of sphericity was violated, Greenhouse–Geisser adjusted *p*-values were reported. Where an interaction effect was found between the streak type and the team, the simple main effect of streak type was assessed on each team separately. Assessing the differences in the demands between teams was beyond the scope of this study. Where no interaction effect was found between the streak type and the team, the streak type main effect was consulted. Pairwise comparisons were conducted using Bonferroni–Holm adjustments. By definition, the shooting percentage in streaks against was always zero, so shooting percentage was only compared between scoring streaks and regular play. Partial eta squared η^2^_p_ effect sizes were calculated for the ANOVA tests, categorized as follows: 0.010–0.039: small, >0.039–0.140: medium, >0.140: large [34]. Cohen’s d effect sizes (ES) for pairwise comparisons were calculated on log-transformed data and categorized as follows: <0.2: trivial, 0.2–0.6: small, >0.6–1.2: moderate, >1.2–2.0: large, >2.0: very large [35].

## 3. Results

The within-participant average Coefficients of Variation for AvF_NET_ and the pooled activity bands were 6.1% and 23.3%, respectively. Time-outs were called by the opposition team more frequently during scoring streaks than during regular play (t_(60)_ = 4.055, *p* < 0.01; median difference (MD) = 0.13 time-outs∙min^−1^; ES = 1.63). The players played 22.7 (9.3–28.2) minutes per match. The individual on-court activity intensity during regular play did not differ for the streak conditions (F_(2,46)_ = 0.99; *p* = 0.34; η^2^_p_ < 0.01), it being approximately 592 N for professional women, 543 N for semi-professional women and 784 N for semi-professional men. The on-court activity intensity distribution was similar across all teams, with approximately 29% of on-court time spent in recovery performing inactive- and light-intensity activity, approximately 45% of on-court time spent performing moderate-vigorous activity and approximately 26% of on-court time spent performing maximal- and supramaximal-intensity activity (Table 2). The teams attempted a shot approximately every 30 s, with 65% of shots being 2-point attempts and an average shooting accuracy of 45%. In regular play, 2-point attempts were shot at 47% (42–54%) accuracy, while 3-point attempts were shot at 33% (29–40%) accuracy. A foul was committed approximately every 2 min, and offensive rebounds were gained at approximately half the frequency of defensive rebounds (Table 3).

The individual on-court activity intensity during scoring streaks, streaks against and regular play for each team separately and for all teams combined is shown in Table 2. The differences between streak types for the majority of activity variables were not dependent on team (streak type x team interaction effect: F_(4,46–54)_ = 0.28–1.93; *p* = 0.16–0.78; η^2^_p_ = 0.03–0.13). When all teams were combined, streak type influenced the proportion of time spent performing moderate-vigorous activity (streak type main effect: F_(2,54)_ = 3.76; *p* = 0.03; η^2^_p_ = 0.12), which was greater during regular play than during streaks against (*p* = 0.05; MD = 2%; ES = 0.28). Streak type did not influence average individual on-court intensity or the proportion of on-court time spent performing inactive, light or supramaximal activity (streak type main effect: F_(4,46–54)_ = 0.50–3.41; *p* = 0.07–0.52; η^2^_p_ = 0.00–0.12).

The differences between streak types for the proportion of on-court time spent performing maximal activity were dependent on team (streak type x team interaction effect: F_(4,54)_ = 3.05; *p* = 0.04; η^2^_p_ = 0.18). For professional women, streak type influenced the proportion of on-court time spent performing maximal activity (streak type main effect: F_(2,20)_ = 6.11; *p* = 0.02; η^2^_p_ = 0.38), with a greater proportion of maximal activity measured during scoring streaks than during streaks against (*p* = 0.05; MD = 1%; ES = 0.79).

The team game-related statistics during scoring streaks, streaks against and regular play for each team separately and for all teams combined are shown in Table 3. The differences in team game-related statistics between streak types were not dependent on team (streak type x team interaction effect: F_(2–4,34–54)_ = 0.71–3.01; *p* = 0.06–0.56; η^2^_p_ = 0.05–0.20), so the main effects for streak type were assessed with all teams together. Streak type influenced the frequency of fouls committed (F_(2,54)_ = 11.55; *p* < 0.01; η^2^_p_ = 0.30). Fouls were committed more frequently during streaks against than during regular play (*p* < 0.01; MD = 0.39∙min^−1^; ES = 1.50) and scoring streaks (*p* < 0.01; MD = 0.77∙min^−1^; ES = 0.96), and they were committed more frequently during regular play than during scoring streaks (*p* < 0.01; MD = 0.38∙min^−1^; ES = 0.83).

Shot selection was dependent on streak type (F_(2,48)_ = 4.08–12.89; *p* < 0.01–0.03; η^2^_p_ = 0.15–0.35). The proportion of shots that were 2-point attempts was lower during streak against than during regular play (*p* < 0.01; MD = 5%; ES = 0.20) and scoring streaks (*p* < 0.01; MD = 15%; ES = 0.34), and the proportion of shots that were 3-point attempts was greater during regular play than during scoring streaks (*p* = 0.01; MD = 10%; ES = 0.26).

The frequency of shot attempts was dependent on streak type (F_(2,54)_ = 58.45; *p* < 0.01; η^2^_p_ = 0.68), with more shots attempted during scoring streaks than during regular play (*p* < 0.01; MD = 1.34∙min^−1^; ES = 1.71) and streaks against (*p* < 0.01; MD = 1.66∙min^−1^; ES = 1.61) and more shots attempted during regular play than during streaks against (*p* < 0.01; MD = 0.33∙min^−1^; ES = 0.94). The teams shot more accurately during scoring streaks than during regular play (*p* < 0.01; MD = 42.44%; ES = 1.87). The proportion of scoring events involving an assist was not different between scoring streaks and regular play (*p* = 0.77).

The frequency of offensive rebounds differed between streak types (F_(2,54)_ = 4.08; *p* = 0.03; η^2^_p_ = 0.13), with offensive rebounds gained more frequently during regular play than during streaks against (*p* < 0.01; MD = 0.34∙min^−1^; ES = 1.10). Streak type also influenced the frequency of defensive rebounds (F_(2,54)_ = 66.16; *p* < 0.01; η^2^_p_ = 0.71), with defensive rebounds gained more frequently during scoring streaks than during regular play (*p* < 0.01; MD = 0.54∙min^−1^; ES = 1.43) and streaks against (*p* < 0.01; MD = 1.32∙min^−1^; ES = 1.70) and such rebounds gained more frequently during regular play than during streaks against (*p* < 0.01; MD = 0.78∙min^−1^; ES = 1.33).

## 4. Discussion

This study used a recently developed accelerometry-derived method for quantifying on-court activity in basketball to provide novel insights into the activity intensities and game-related statistics associated with team-based scoring streaks. Individual on-court activity intensity was generally consistent between streak types, with a slightly greater proportion of moderate-vigorous (all teams combined) and maximal (professional women) activity undertaken during streaks against when compared to regular play. During scoring streaks, 3-point shots were attempted less frequently than during regular play, while the volume of shot attempts, shooting percentage and defensive rebounds were also greater than during regular play.

The average individual on-court activity intensity was not different between streak types, and few differences existed between streaks and regular play for the relative exercise intensity bands. Specifically, the 2% lower proportion of moderate-vigorous activity might be either a cause or effect of reduced performance, and at the professional women’s level, the 1% greater proportion of maximal activity might be either a cause or effect of better performance. It is possible that less activity in these exercise intensity categories was a catalyst for poorer performance during streaks against; however it is also possible that being in a streak against reduced the team’s energy and motivation [36], causing their moderate-vigorous and maximal intensity activity to decrease. Previous research shows that differences in on-court activity intensity between wins and losses are inconsistent between studies, with more high-intensity activity during losses [7], more high-intensity activity during wins [9] and no differences in activity between good and bad performance [6] all having been found. The inconsistencies in the previous findings combined with the limited differences in the present study suggest that individual on-court activity intensity might not be a dominant factor related to either overall or transient success in basketball. The lack of differences in activity intensity paired with the multiple differences in game-related statistics also suggests that changes in strategy might not always elicit changes in activity intensity. During streaks against, coaches can encourage their players to maintain their normal activity intensity, substitute some rested players into the match or call a time-out to give the on-court players recovery time; however, it is likely that more benefit will be gained from addressing game-related strategies, such as shot attempts and rebounds.

Shot selection differed between streaks and regular play (large effect), with 10% fewer 3-point shots attempted scoring streaks compared to regular play and 5–15% fewer 2-point shots attempted during streaks against compared to regular play and scoring streaks, where the shot-type ratio of 3:1 2-point to 3-point attempts was associated with scoring streaks. It is logical that a greater proportion of 2-point attempts is associated with more scoring success because 2-point attempts are shot at a higher success rate than 3-point attempts [37,38]. To enable the higher proportion of 2-point attempts in scoring streaks, it is possible that the opposition’s defence was not performing optimally, allowing the streaking team to attack inside the 3-point line, or it is possible that the streaking team’s offence was superior and able to penetrate the opposition’s defence more effectively. Alternatively, a combination of the two is possible [39]. During periods of streaks against, it is also possible that a reduced tendency to attempt safer 2-point shots led to more missed shots, allowing the opposition to score consecutively. Previous researchers have suggested that scoring momentum in sport is associated with changes in motivation, perceptions of control, optimism, energy and team synchronization [36], which might contribute to the differences in shot attempts between scoring streaks, streaks against and regular play. For example, it is possible that when teams were in a scoring streak, they felt in control and confident in their offence and therefore felt that they could reliably generate 2-point shot attempts rather than needing to attempt riskier 3-point shots. To maximize the chances of scoring streaks, teams should aim for approximately three quarters of their shot attempts to be 2-point attempts. When a team is experiencing a streak against, on defence, they should focus on forcing the opposition to shoot from outside the 3-point line, and on offence, they should aim to generate shooting options close to the basket rather than taking riskier 3-point attempts, despite the greater reward available for a successful 3-point attempt.

One extra defensive rebound was gained every two minutes during scoring streaks than during regular play (large effect), while both offensive and defensive rebounds were lower during streaks against than during regular play (large effects). A higher frequency of defensive rebounds during scoring streaks is expected, as the opposition team missing all their shots provides the streaking team with more opportunity for defensive rebounds. Additionally, fewer defensive rebounds during streaks against are facilitated by the streaking team shooting more successfully compared to regular play, meaning the team experiencing the streak against has less opportunity for defensive rebounds. Interestingly, when a team experiencing a streak against, they are missing all the shots that they take, which theoretically provides them with more opportunity for offensive rebounds; however, offensive rebounds were also lower during streaks against compared to regular play. This finding suggests that an inability to maintain possession through offensive rebounds might contribute to streaks occurring. It has been identified that more defensive rebounds are associated with more overall match success when comparing wins and losses [1,2,3,4], and fewer offensive rebounds are associated with less overall match success [1,2]. The findings of the present study support the notion that maximizing opportunities to regain possession through defensive rebounds and maintain possession through offensive rebounds is important for success. During streaks against, teams might choose to emphasize positioning for rebounds at both ends of the court to slow the opposition team’s scoring momentum. When aiming to optimize rebounding opportunities, it is important that players do not increase their frequency of fouls committed, as more fouls were committed during streaks against (large effect), and fewer fouls were committed during scoring streaks (moderate effect), than during regular play.

Scoring streaks were associated with both 1.3 more shot attempts per minute and a 43% greater shooting percentage compared to regular play (large effects), while streaks against were associated with 0.3 fewer shot attempts per minute compared to regular play (moderate effect). These findings demonstrate that a combination of both an increased volume of shot attempts and the increased accuracy of those shot attempts is related to success. Fewer shot attempts during streaks against suggest that the team is frequently losing possession before they attempt a shot. Fewer shot attempts might also be caused by the team experiencing the streak against getting fewer rebounds, as highlighted previously, meaning they have less opportunity to attempt shots. During scoring streaks, shot selection likely influenced shooting percentage, as a greater proportion of 2-point attempts during scoring streaks means that those shots have a greater chance of being successful. The results from previous studies show that greater field goal shooting percentage is associated with winning team results [1,2,4]. The findings from the present study suggest that, to facilitate the occurrence of scoring streaks, teams need to both attempt a greater number of shots and shoot at a greater percentage. In order to achieve this outcome, teams can focus on attempting more 2-point shots to improve their shooting percentage.

While this study presents novel findings on the factors related to scoring streaks in basketball, some limitations are present. It is acknowledged that these findings might not be generalizable to teams outside those included in this study. However, given that three teams were included across multiple genders and competition levels and that the team was typically not a significant interacting factor, these findings are more generalizable than if only one team had been used, as is typical for research within elite sport [6,7,9]. We also acknowledge that positioning the accelerometer closer to the players’ center of mass would have been preferable; however, player safety and comfort was prioritized when deciding where to position the accelerometers. The accelerometer position of between the scapulae is consistent with previous work [23,24]. It is also possible that the minimum scoring rate of one scoring event every 36 s in the scoring streak definition either over- or under-estimated the occurrence of the type of scoring streaks we were aiming to quantify. Additionally, while associations between scoring streaks and various game-related statistics and time-motion analysis variables were found, it is not possible to determine whether the on-court behavior influenced the occurrence of streaks or if the occurrence of streaks influenced the on-court behavior. Future research could focus on identifying possible catalysts and mitigators for streaks occurring by assessing the game-related statistics and time-motion analysis variables leading into the beginning of a streak and following the end of a streak. Future research could also investigate alternative factors that might contribute to streaks occurring, such as on-court team composition or offensive and defensive strategies. Lastly, while this study investigated scoring streaks and changes of momentum within individual matches, this concept could be progressed through the investigation of performance momentum across multiple matches during a season.

## 5. Conclusions

Using accelerometers to quantify on-court activity in basketball, it was determined that maintaining moderate-vigorous (for all teams) and maximal (for professional women) intensity activity might contribute to mitigating the chances of streaks against occurring in basketball. The practical implications for coaches include: (1) to maximize the probability of scoring streaks, teams should aim to shoot at a 3:1 2-point to 3-point ratio, which can facilitate a greater shooting percentage; (2) teams should also focus on winning defensive rebounds without sacrificing fouls, which can facilitate a greater volume of shot attempts; (3) to limit the probability of streaks against, teams should focus on generating 2-point shot attempts despite the greater reward for successful 3-point attempts; and (4) teams should also focus on maintaining possession through offensive rebounds and, where possible, regaining possession through defensive rebounds to facilitate a greater volume of shot attempts.

## Figures and Tables

**Table 1:** Participant characteristics.

Team	Players	Age (years)	Stature (cm)	Mass (kg)	Matches Monitored	Scoring Streaks	Proportion of Match Time Spent in Scoring Streaks (%)	Streaks Against	Proportion of Match Time Spent in Streaks Against (%)
P Women	12	25.2 ± 5.9	181 ± 11	79.3 ± 17.1	20	15	2.2	31	4.5
SP Women	12	28.1 ± 5.0	176 ± 10	75.9 ± 18.2	20	31	4.8	15	2.3
SP Men	13	26.8 ± 5.2	192 ± 8	96.2 ± 16.4	22	41	6.0	26	3.7

SP = semi-professional, P = professional.

**Table 2:** On-court activity intensity during scoring streaks, streaks against and regular play for each team and for all teams combined.

	Professional Women			Semi-Professional Women			Semi-Professional Men			All Teams Together		
	Scoring Streaks	Regular Play	Streaks Against	Scoring Streaks	Regular Play	Streaks Against	Scoring Streaks	Regular Play	Streaks Against	Scoring Streaks	Regular Play	Streaks Against
AvF_NET_	579 (550–618)	592 (547–635)	600 (550–654)	554 (522–627)	543 (512–580)	549 (515–595)	808 (713–848)	784 (718–845)	782 (727–843)	618 (554–750)	608 (547–760)	627 (550–779)
Inactive (%)	5.9 (3.9–12.2)	6.6 (4.8–8.6)	6.0 (4.4–9.9)	6.8 (0.7–18.1)	5.5 (4.1–8.4)	8.5 (4.2–15.3)	6.6 (3.1–9.9)	6.3 (4.9–9.5)	5.7 (3.4–8.5)	6.6 (3.2–11.2)	6.3 (4.8–8.5)	6.3 (4.4–10.3)
Light (%)	23.4 (18.0–28.9)	22.2 (19.2–24.6)	20.8 (19.1–22.5)	21.5 (14.3–40.8)	19.4 (16.7–27.6)	20.3 (13.9–22.3)	22.3 (19.1–28.4)	23.5 (18.5–26.7)	26.3 (18.5–29.0)	22.3 (17.8–29.1)	22.6 (18.2–25.7)	20.8 (18.6–25.8)
Moderate-Vigorous (%)	41.8 (36.0–45.7)	40.2 (35.9–49.1)	42.1 (35.5–48.7)	39.9 (31.0–46.6)	43.4 (39.1–46.8)	40.6 (30.1–46.2)	46.3 (38.3–49.4)	46.5 (39.3–50.5)	45.2 (37.4–51.1)	42.1 (36.6–48.2)	44.5 (38.9–49.1)	43.4 ^ (35.4–48.7)
Maximal (%)	12.4 (10.2–14.5)	10.7 (9.8–12.0)	10.6 * (10.2–11.9)	9.2 (6.7–12.1)	12.6 (9.4–15.9)	9.1 (8.5–12.7)	8.7 (7.6–12.5)	9.8 (7.8–12.5)	8.9 (7.4–10.6)	10.3 (8.2–13.0)	10.3 (9.2–12.6)	10.2 (8.2–11.6)
Supramaximal (%)	14.7 (8.8–21.2)	15.1 (9.7–25.0)	15.2 (11.8–29.4)	14.6 (12.7–28.6)	18.9 (11.3–20.4)	20.4 (10.8–27.9)	14.2 (9.9–16.5)	15.0 (11.9–16.1)	14.1 (11.0–20.2)	14.6 (11.4–18.3)	15.3 (11.1–19.9)	14.7 (11.6–24.6)

* Significantly different to scoring streaks, ^ significantly different to regular play. Values presented as median (lower quartile–upper quartile).

**Table 3:** Game-related statistics during scoring streaks, streaks against and regular play for each team and for all teams.

	Professional Women			Semi-Professional Women			Semi-Professional Men			All Teams Together		
	Scoring Streaks	Regular Play	Streaks Against	Scoring Streaks	Regular Play	Streaks Against	Scoring Streaks	Regular Play	Streaks Against	Scoring Streaks	Regular Play	Streaks Against
Fouls∙min^−1^	0.0 (0.0–0.9)	0.4 (0.3–0.4)	0.6 (0.0–1.8)	0.0 (0.0–0.1)	0.3 (0.2–0.4)	0.5 (0.4–1.5)	0.0 (0.0–0.3)	0.4 (0.4–0.5)	1.2 (0.6–3.6)	0.0 ^ (0.0–0.3)	0.4 * (0.3–0.5)	0.8 *^,^^ (0.2–1.9)
Proportion of shot attempts that were 2P attempts (%)	67 (50–93)	67 (63–71)	71 (33–100)	80 (65–95)	70 (65–78)	25 (0–50)	70 (60–75)	60 (55–64)	61 (33–67)	75 (62–89)	65 (59–71)	60 *^,^^ (0–75)
Proportion of shot attempts that were 3P attempts (%)	33 (7–50)	33 (29–37)	29 (0–67)	20 (5–35)	30 (22–35)	75 (50–100)	30 (25–40)	40 (36–45)	39 (33–67)	25 ^ (11–38)	35 * (29–41)	40 (25–100)
Offensive rebounds∙min^−1^	0.0 (0.0–0.2)	0.3 (0.2–0.4)	0.0 (0.0–0.1)	0.0 (0.0–0.5)	0.4 (0.3–0.4)	0.4 (0.0–0.5)	0.4 (0.0–0.6)	0.3 (0.2–0.4)	0.0 (0.0–0.0)	0.0 (0.0–0.5)	0.3 (0.3–0.4)	0.0^ (0.0–0.4)
Defensive rebounds∙min^−1^	1.3 (1.0–1.8)	0.7 (0.7–0.8)	0.0 (0.0–0.3)	1.2 (0.9–1.5)	0.8 (0.7–0.8)	0.0 (0.0–0.4)	1.4 (1.1–1.5)	0.7 (0.6–0.9)	0.0 (0.0–0.1)	1.3 ^ (1.0–1.6)	0.8 * (0.7–0.8)	0.0 *^,^^ (0.0–0.2)
Shot attempts∙min^−1^	3.1 (2.7–3.6)	1.8 (1.7–2.0)	1.4 (1.1–1.9)	3.1 (2.7–3.9)	2.0 (1.7–2.1)	1.7 (1.0–2.1)	3.4 (2.9–4.0)	1.9 (1.8–2.0)	1.5 (0.5–1.9)	3.2 ^ (2.8–3.9)	1.9 * (1.7–2.0)	1.6 *^,^^ (1.1–1.9)
Combined shooting percentage (field goals and free throws combined)	100 (94–100)	44 (42–49)	0 ^#^	87 (78–100)	46 (42–51)	0 ^#^	86 (75–92)	46 (44–50)	0 ^#^	88 ^ (79–100)	45 * (42–50)	0 ^#^
Proportion of scoring shots involving an assist (%)	50 (42–67)	60 (52–69)	-	80 (67–95)	78 (67–83)	-	50 (33–63)	54 (40–67)	-	60 (50–80)	60 (50–70)	-

* Significantly different to scoring streaks, ^ significantly different to regular play, ^#^ by definition, 2P 2-point shots, 3P 3-point shots. Values presented as median (lower quartile–upper quartile).

## Data Availability

The data presented in this study are available on request from the corresponding author. The data are not publicly available due to privacy.
